# DNA Methylation Landscapes of Human Fetal Development

**DOI:** 10.1371/journal.pgen.1005583

**Published:** 2015-10-22

**Authors:** Roderick C. Slieker, Matthias S. Roost, Liesbeth van Iperen, H. Eka D. Suchiman, Elmar W. Tobi, Françoise Carlotti, Eelco J. P. de Koning, P. Eline Slagboom, Bastiaan T. Heijmans, Susana M. Chuva de Sousa Lopes

**Affiliations:** 1 Molecular Epidemiology Section, Leiden University Medical Center, Leiden, The Netherlands; 2 Department of Anatomy and Embryology, Leiden University Medical Center, Leiden, The Netherlands; 3 Department of Nephrology, Leiden University Medical Center, Leiden, The Netherlands; 4 Hubrecht Institute, Utrecht, The Netherlands; 5 Department for Reproductive Medicine, Ghent University Hospital, Ghent, Belgium; The Babraham Institute, UNITED KINGDOM

## Abstract

Remodelling the methylome is a hallmark of mammalian development and cell differentiation. However, current knowledge of DNA methylation dynamics in human tissue specification and organ development largely stems from the extrapolation of studies *in vitro* and animal models. Here, we report on the DNA methylation landscape using the 450k array of four human tissues (amnion, muscle, adrenal and pancreas) during the first and second trimester of gestation (9,18 and 22 weeks). We show that a tissue-specific signature, constituted by tissue-specific hypomethylated CpG sites, was already present at 9 weeks of gestation (W9). Furthermore, we report large-scale remodelling of DNA methylation from W9 to W22. Gain of DNA methylation preferentially occurred near genes involved in general developmental processes, whereas loss of DNA methylation mapped to genes with tissue-specific functions. Dynamic DNA methylation was associated with enhancers, but not promoters. Comparison of our data with external fetal adrenal, brain and liver revealed striking similarities in the trajectory of DNA methylation during fetal development. The analysis of gene expression data indicated that dynamic DNA methylation was associated with the progressive repression of developmental programs and the activation of genes involved in tissue-specific processes. The DNA methylation landscape of human fetal development provides insight into regulatory elements that guide tissue specification and lead to organ functionality.

## Author Summary

Methylation of DNA is a key epigenetic mark. Adult tissues have highly distinct genome-wide DNA methylation signatures. How these signatures arise during human fetal development is largely unknown. Here, we studied DNA methylation profiles of four tissues (amnion, muscle, adrenal, pancreas) during first and second trimester of human fetal development. Already in the first trimester, a tissue-specific signature was found in each of the tissues. However, during the first and second trimester, a substantial number of genomic regions were found to gain and lose DNA methylation. Genomic regions that gained methylation were associated with the shut-down of developmental processes, while genomic regions that lose methylation were associated with the activation of tissue-specific functions. These findings on the DNA methylation landscape of human fetal development are important as they provide insight into regulatory elements that guide tissue specification and lead to organ functionality.

## Introduction

Methylation of CpG dinucleotides in the mammalian genome is a key epigenetic mark. Adult tissues have highly distinct genome-wide DNA methylation signatures consistent with the regulation of cell differentiation by epigenetic mechanisms [1–3]. Differences in DNA methylation between tissues have been shown to mark differences between germ layers [4], preferentially at regions with low CpG content [2,5,6], at enhancers [4] and alternative promoters [7,8].

Multiple studies have reported on the reprogramming of the human methylome during preimplantation embryo development [9–11]. In line with previous data on mice [12], in humans DNA methylation is largely erased after conception, the paternal genome being actively and the maternal genome passively demethylated, to become remethylated with the implantation of the embryo [9,10,13,14]. However, systematic and detailed reports on DNA methylation dynamics during human fetal development remain scarce [15], while such data is key to understand how epigenetic mechanisms drive tissue specification and organ functionality. Current views of fetal DNA methylation dynamics are largely extrapolated from studies on the differentiation of human and mouse cells *in vitro* [7,15–21], and the comparison of differentiated tissues to human induced pluripotent stem cells and human embryonic stem cell lines [15]. An exception is fetal brain development in humans, for which recently reported *in vivo* data showed significant DNA methylation remodelling [15,22].

Recent developments in technology for interrogating genome-wide DNA methylation at single-nucleotide resolution [23] and detailed functional annotation of the human genome [24,25] provide an opportunity to chart DNA methylation during development and assign biological roles to the regions involved. Taking advantage of these developments, we report on DNA methylation dynamics during human fetal development of one extraembryonic tissue and three organs relevant for complex human diseases. This organ-specific catalogue of DNA methylation during development provides fundamental insights into processes guiding human development, but also into the biological function of non-coding regions, which are emerging as important from genome-wide association studies (GWASs) of complex diseases [26]. In addition, this catalogue may serve as a reference for studies on the role of epigenetic mechanisms in the association between an adverse prenatal environment and adulthood disease [27] since DNA methylation marks may have an heightened sensitivity for environmental perturbations during remodelling [28].

## Results

### Fetal DNA methylation reflects tissue origin and developmental age

To study DNA methylation dynamics in human fetal development, amnion, muscle, adrenal and pancreas samples of 11 fetuses were obtained at 9, 18 and 22 weeks of gestation (W9, W18 and W22; S1A Fig). Genome-wide DNA methylation was investigated with the Illumina 450k array resulting in data on 452,490 CpG sites after quality control [29] (S1B–S1E Fig). The study included three biological replicates per tissue and time point, except for W22 amnion (n = 2) and W22 pancreas (n = 2) (S2A Fig).

**Fig 1:**
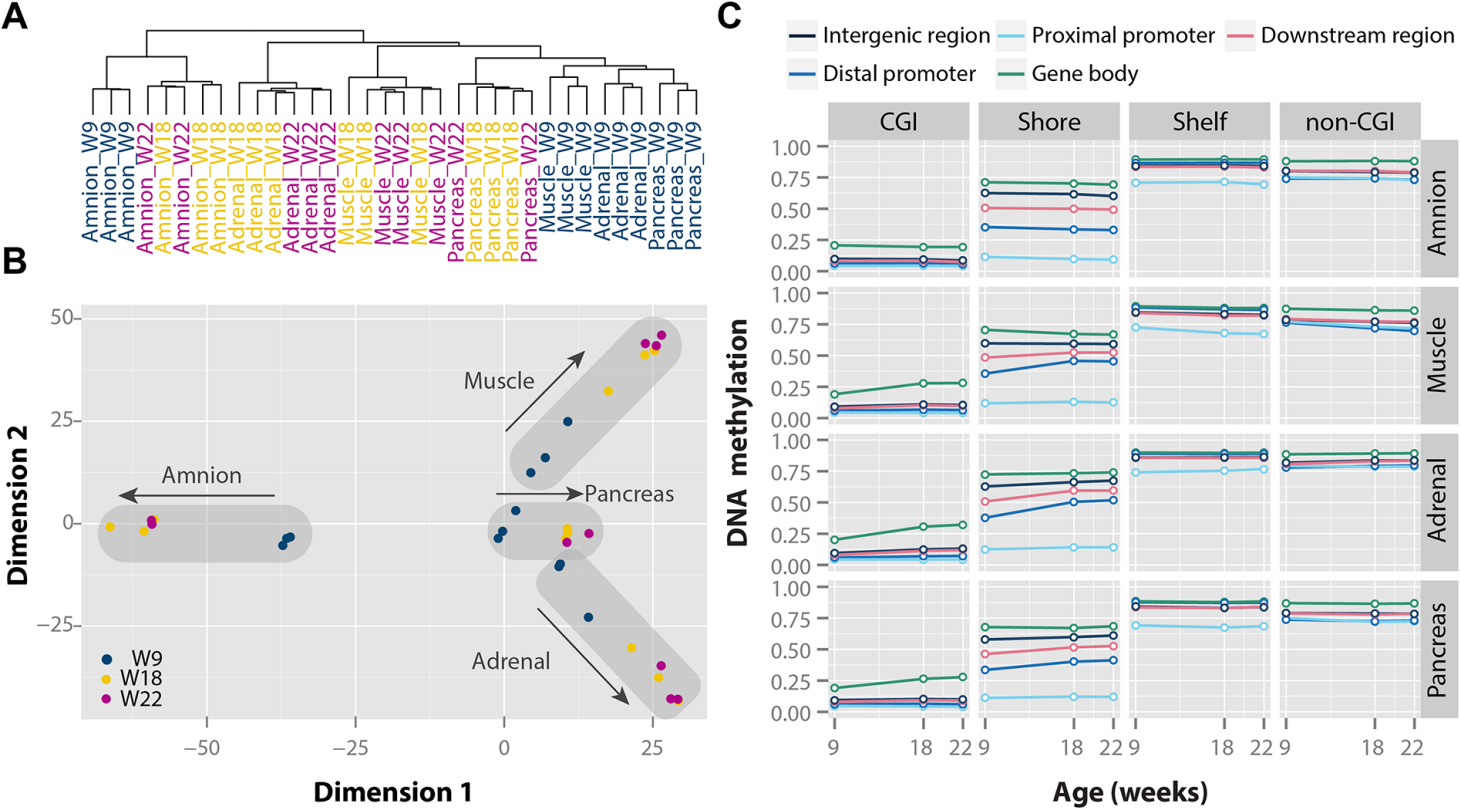
Tissue- and time-specific DNA methylation patterns during human fetal development. (**A**) Clustering based on Euclidean distance. (**B**) Multidimensional scaling based on Euclidean distance of the four tissues. (**C**) Median DNA methylation for each of the four tissues over time with a combined genic and CGI-centric annotation. CGI, CpG island.

We first assessed differences in overall DNA methylation patterns between time points and tissues using hierarchical clustering based on Euclidean distance (Fig 1A) and multidimensional scaling (MDS) (Figs 1B and S2B). DNA methylation patterns clearly differentiated the four tissue types studied (Figs 1A, 1B and S2B). The amnion, representing an extraembryonic tissue, clustered separately from the three embryonic tissues (Fig 1A). Within the embryonic cluster, all W9 tissues (representative of the first trimester) clustered together, whereas W18-W22 tissues (representative of the second trimester) were present towards the edges of the MDS plot (Figs 1B and S2B). Despite the distinct differences in DNA methylation patterns from the first to second trimester, the total number of hypo-, intermediately and hypermethylated CpGs remained constant across time points and tissues (including the extraembryonic amnion) on both autosomes and, in females, the X chromosome (S2C and S2D Fig). This suggests that the observed differences in the MDS plot were not driven by changes in average levels of DNA methylation, but rather due to tissue- and time-specific changes in DNA methylation.

To validate our findings, we integrated our data with three previously published Illumina 450k datasets on 10 human fetal tissues [15,22,30]. Hierarchical clustering of all data together (n = 117) confirmed the presence of distinct tissue- and time-specific DNA methylation patterns in fetal tissues (S2E Fig).

The characteristics and biological function of DNA methylation depend on the local CpG content and position relative to genes [31]. We mapped CpG sites (CpGs) to CpG islands (CGIs as defined in the UCSC genome browser; 138,919 CpGs), their shores (±2 kb of CGIs; 103,453 CpGs) and shelves (±2 kb of shores; 42,227 CpGs), and remaining CpG-poor non-CGI regions (157,560 CpGs), and to regions relative to gene locations including distal promoters (-10 kb–-1.5 kb; 21,101 CpGs), proximal promoters (-1.5 kb–+0.5 kb; 171,077 CpGs), gene bodies (+0.5 kb–3’ untranslated region (UTR); 175,062 CpGs), downstream regions (3’ UTR–+5 kb; 8,563 CpGs) and remaining intergenic regions (66,356 CpGs; Fig 1C). CpGs were commonly hypomethylated in CGIs, intermediately methylated in shores and hypermethylated in both shelves and non-CGI regions (Fig 1C). These patterns differed by genic position, e.g. CGI methylation was lowest in proximal promoters and highest in gene bodies. Annotation-specific methylation differences were found between W9 and W22, as CpGs in CGIs and shores tended to increase (e.g. gene body CGIs and distal promoter shores), whereas CpGs in non-CGI regions decreased in methylation (e.g. non-CGI promoters). For a subset of annotations, the amnion showed a slightly different DNA methylation patterns than for embryonic tissues, e.g. for gene body CGIs (Fig 1C). Taken together, our data imply that DNA methylation is highly dynamic during fetal development without affecting the average level of DNA methylation.

### Hypomethylation discriminates tissues independent of developmental age

**Fig 2:**
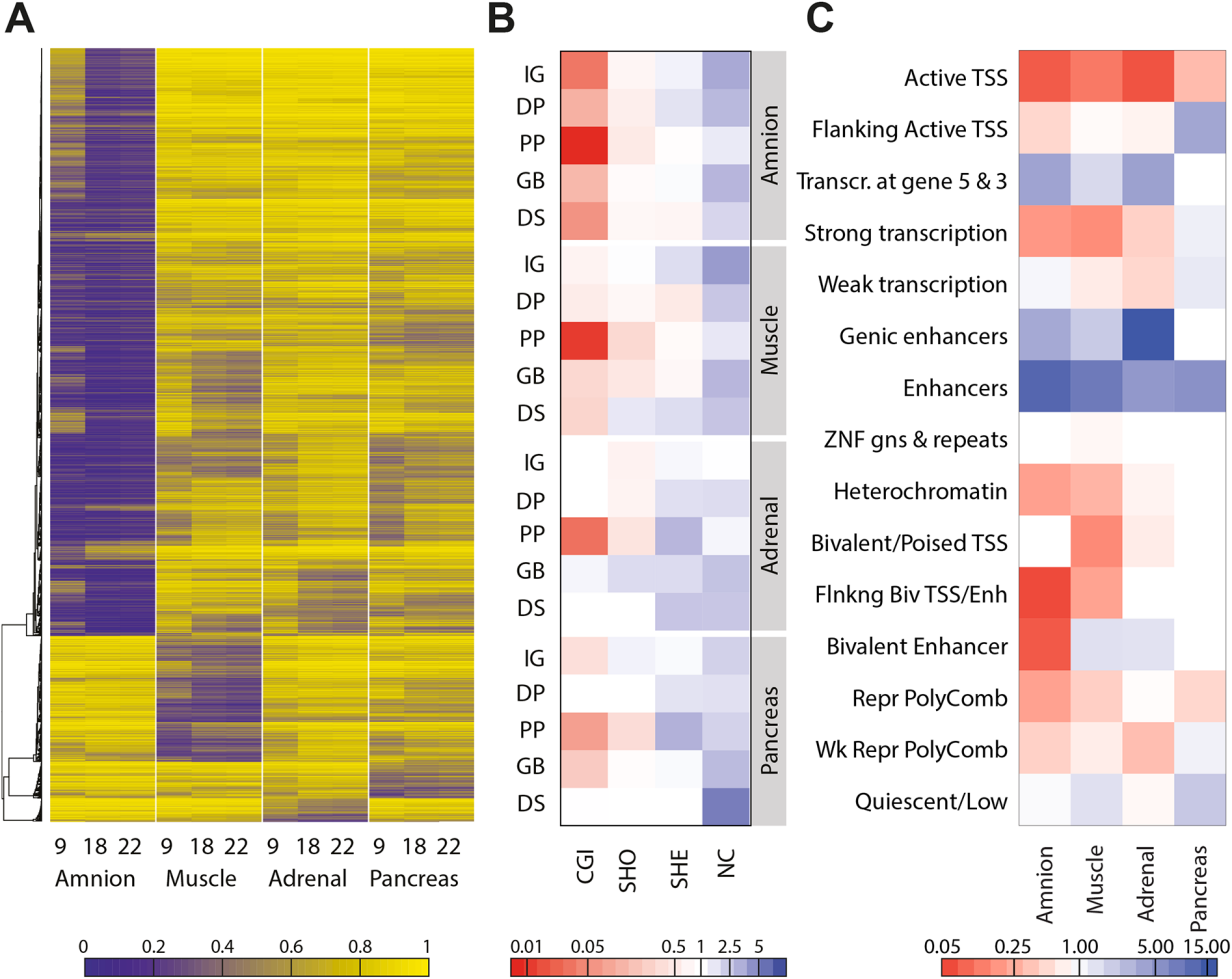
Sets of hypomethylated CpG sites are tissue-specific. (**A**) Heatmap representing hypomethylated CpGs per tissue, defined by a beta difference of ≥ 0.2 of the studied tissue compared to the other tissues. (**B)** Combined genic and CGI-centric annotation for the hypomethylated CpGs per tissue represented as the odds ratio (see S3A Fig for odds ratios). CGI, CpG island; DP, distal promoter; DS, downstream; GB, gene body; IG, intergenic; NC, non-CGI; PP, proximal promoter; SHE, shelves; SHO, shores. (**C**) Enrichment of hypomethylated CpGs in the chromatin state segmentation states for the matching tissues (amnion, fetal muscle, fetal adrenal and adult pancreatic islets; see S3B Fig for odds ratios).

It has been shown that each adult tissue is defined by tissue-specific DNA hypomethylation [15,32,33]. Since the four fetal tissues analysed showed a clear DNA methylation signature that corresponded to separated clusters (Fig 1A and 1B), we investigated whether combinations of tissue-specific DNA hypomethylated CpGs were present irrespective of its developmental stage. To do this, we identified CpGs that were relatively hypomethylated (defined as a DNA methylation difference of > 20%) in each tissue compared to all others throughout the three time points of fetal development investigated. The analysis showed indeed that, independently of the developmental age, each tissue showed a cluster of tissue-specific hypomethylated CpGs (Fig 2A). The early lineage segregation of the amnion was further confirmed by the comparatively large number of CpGs (3,536 CpGs) that were exclusively hypomethylated across amniotic samples. In contrast, the embryonic tissues contained much fewer tissue-specific hypomethylated CpGs (muscle 756 CpGs; adrenal 140 CpGs; pancreas 220 CpGs) reflecting their common origin of the epiblast, that gives rise to all embryonic tissues. Genes mapping (i.e. the nearest gene locus) to the specific hypomethylated CpGs per tissue regardless of the time point (amnion 2372, muscle 548, adrenal 120, pancreas 175) were enriched for biological processes that included GO terms characteristic of amnion, muscle and pancreas development and function (S1 Table).

When annotated to genic and CGI-related location, it became evident that tissue-specific hypomethylation was enriched for non-CGI regions (*P* < 0.0001) and highly depleted at CGIs, in particular when mapping to proximal promoters (*P* < 0.0001; Figs 2B and S3A). To gain further insight in the biological role of genomic regions displaying tissue-specific hypomethylation, we used chromatin state segmentations for fetal muscle, fetal adrenal, amnion and adult pancreatic islets generated by the Epigenomics Roadmap [25]. Tissue-specific hypomethylation was strongly enriched at enhancers (*P* < 0.001; Figs 2C and S3B). The functional relevance of those tissue-specific hypomethylated CpGs was further validated by comparison to additional fetal samples [15] and adult somatic tissues [6] from available external datasets (S3C Fig). Intriguingly, a high degree of similarity between tissues sharing the same origin, even into adulthood, was observed (S3C Fig).

We next investigated whether tissue-specific hypomethylated CpGs clustered into Hypomethylated Regions (tHRs, defined as 3 consecutive hypomethylated CpGs within 1kb of each other) [6]. This was the case for amnion, muscle and pancreas (Tables 1 and S2). tHRs comprise robust development-independent epigenetic markers as exemplified by the mapping of pancreatic tHRs to proximal promoters of the nearest genes *ACY3*, *HNF1A*, and *HNF4A* (Table 1 and S3D Fig), genes with a key role in pancreas development [34,35]. Muscle tHRs (S2 Table) mapped to distal elements of transcription factors involved in muscle development (*NFATC1*) and somitogenesis (*UNCX*) [36,37] (S3D Fig and Tables 1 and S2). Importantly, we could confirm the tHRs identified with the relatively sparse Illumina 450k array with fetal and adult muscle whole-genome bisulfite sequencing (WGBS) data (S3E Fig) [25]. These data indicate that it is feasible to use combinations of tHRs as tissue-specific and development-independent barcodes.

**Table 1:** Table with the numbers of tHRs and three representative genes per tissue associated with tHRs. SM, skeletal muscle; TF, transcription factor.

Tissue	Gene	Feature	Function
**Amnion**	*SLC22A2*	PP	Tubular uptake of organic compounds from circulation
(67 tHRs)	*VTCN1*	PP	B7 costimulatory protein family
	*SLC39A2*	PP	Zinc, iron, and calcium homeostasis
**Muscle**	*UNCX*	IG	TF involved in somitogenesis and neurogenesis
(15 tHRs)	*NFATC1*	IG	Involved in SM development/differentiation
	*DPT*	PP	Extracellular structure organization
**Pancreas**	*ACY3*	PP	Aminoacylase activity
(3 tHRs)	*HNF1A*	PP	Transcriptional activator
	*HNF4A*	PP	Transcriptional activator

### Distinct roles for gain and loss of DNA methylation in fetal development

**Fig 3:**
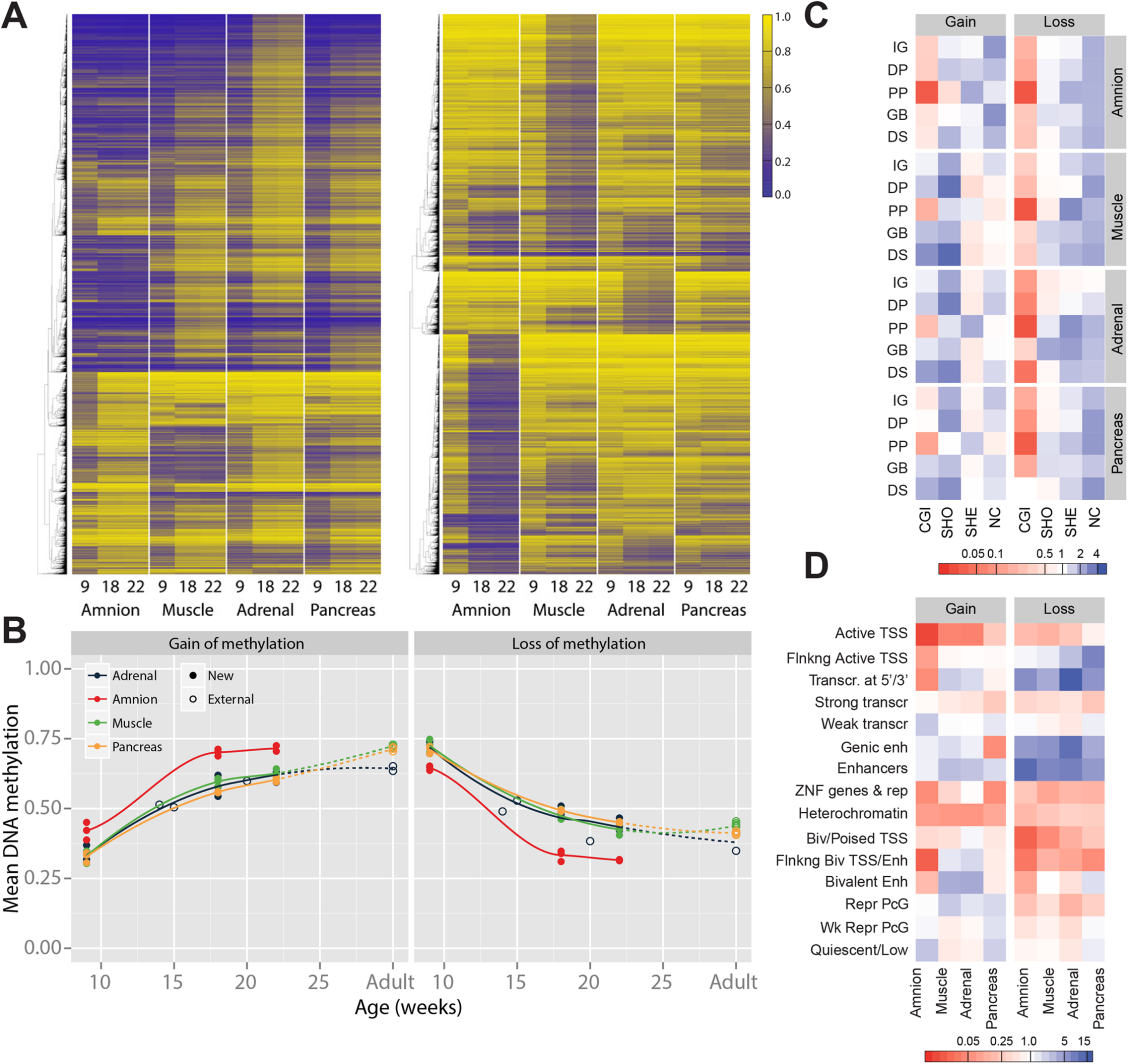
Gain and loss of DNA methylation during human fetal development. (**A**) Heatmap of CpGs with a gain and a loss, respectively, of methylation over time. Gain and loss of methylation was defined as a difference of beta ≥ 0.2 between W9 and W22, and W18 in between. (**B**) Mean methylation of CpGs with a gain or loss of DNA methylation for fetal tissues and their adult counterpart. (**C)** Combined genic and CGI-centric annotation for CpGs with a gain or a loss of methylation represented as the odds ratio (see S4B Fig for odds ratios). CGI, CpG island; DP, distal promoter; DS, downstream; GB, gene body; IG, intergenic; NC, non-CGI; PP, proximal promoter; SHE, shelves; SHO, shores. (**D**) Enrichment of dynamically methylated CpGs in the chromatin segmentation states for the matching tissues (fetal muscle, fetal adrenal, amnion and adult pancreatic islets; see S4C Fig for odds ratios).

We provide evidence for large-scale DNA methylation dynamics between W9 and W22 (DNA methylation difference > 20%) that affected 11.5% of evaluated CpGs (52,134/452,490). Approximately equal numbers of CpGs showed a gain of methylation (GOM) (26,555 CpGs; amnion 5,988; muscle 7,631; adrenal 13,997; pancreas 8,620) and a loss of methylation (LOM) (25,579 CpGs; amnion 10,811; muscle 11,925; adrenal 4,476; pancreas 3,286) (Fig 3A). DNA methylation remodelling occurred predominantly between W9 and W18 and not between W18 and adulthood (Figs 3B and S4A). Intriguingly, the integration and re-analysis of external DNA methylation data of fetal adrenal, brain and liver [6,15,22,30,38] revealed a striking confirmation of DNA methylation dynamics during fetal development. Furthermore, for all embryonic tissues, the DNA methylation levels at W22 were similar to those found in the adult counterpart (Figs 3B and S4A), suggesting that the extent of changes after W22 are limited for these CpGs.

GOM CpGs did not show tissue-specific patterns (Fig 3A) and, in line with this observation, often corresponded to genes involved in generic developmental and cellular processes, including embryonic morphogenesis and regulation of transcription (S3 Table). In contrast, the LOM CpGs were highly tissue-specific (Fig 3A) and mapped to genes involved in tissue-specific processes that matched the organ in which the LOM CpGs were identified (S3 Table). CpGs that lost methylation in the amnion mapped, amongst others, to genes that were associated with the regulation of apoptosis and cytoskeleton organization; in the muscle to genes associated with cytoskeleton organization and muscle system processes; in the adrenal to genes associated with regulation of macromolecule metabolism (S3 Table). In the pancreas no significant enrichments were found.

GOM and LOM CpGs differed in their genomic annotation. While GOM CpGs were generally enriched in CGIs and CGI-shores, LOM CpGs were enriched for CGI-shelves and non-CGI regions (Figs 3C and S4B). LOM- and GOM-specific enrichments were also observed for Epigenomics Roadmap chromatin state segmentations. LOM CpGs were strongly enriched for (genic) enhancers and transcribed regions, whereas GOM CpGs were enriched for bivalent and repressed regions and only modestly at enhancers (Figs 3D and S4C). The results underscore the relevance of DNA methylation in enhancer activity, in addition to the well-studied relationship between DNA methylation and promoter activity [39].

**Fig 4:**
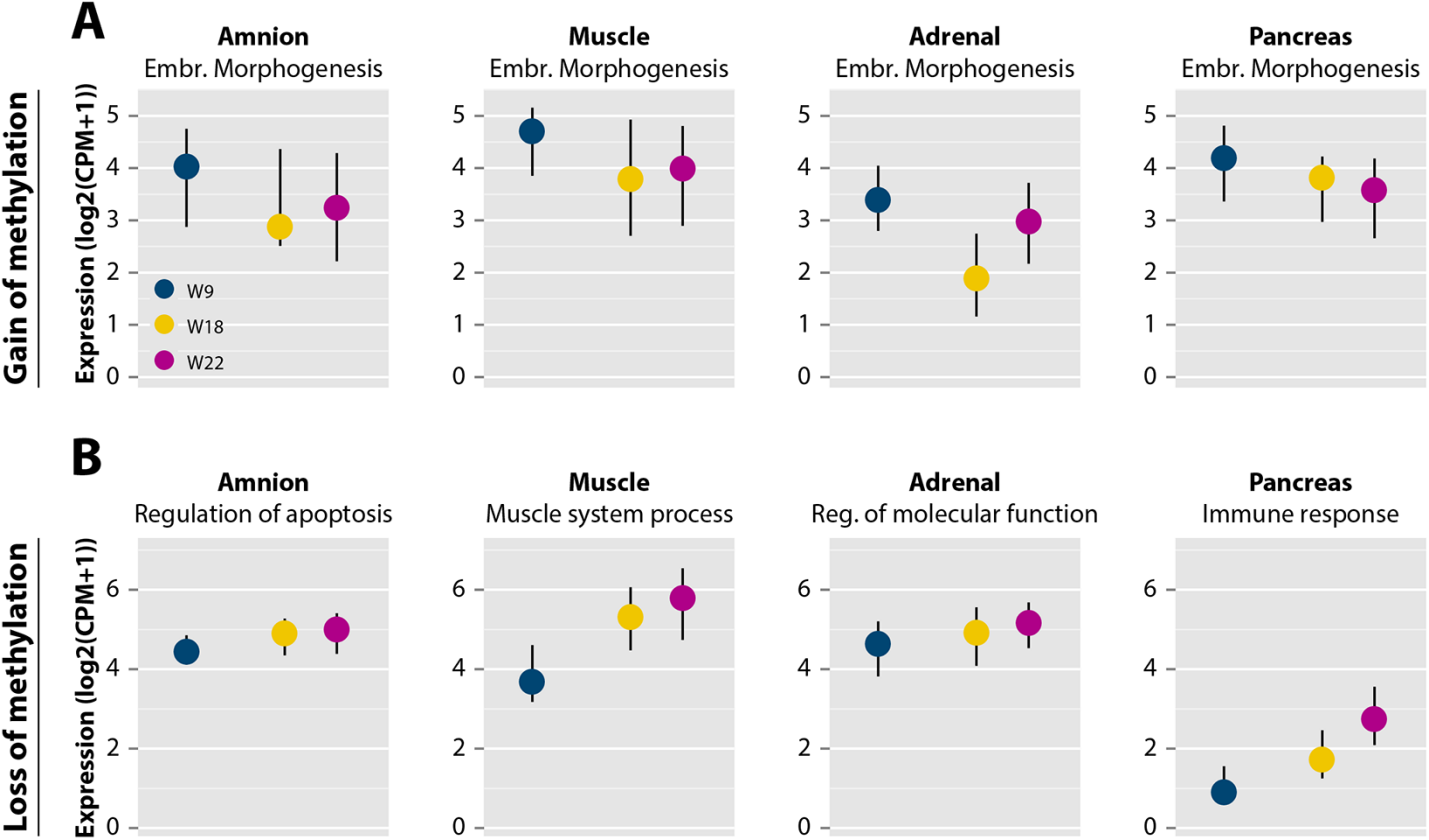
DNA methylation dynamics are accompanied by changes in gene expression. (**A**) Expression profiles of genes in embryonic morphogenesis near dynamic regions with gain of methylation represented as median with the interquartile range (IQR) [40]. (**B**) Expression profiles of genes near dynamic regions with loss of methylation grouped by significant, tissue-specific Gene Ontology terms for each of the four tissues from S3 Table represented as median with IQR [40]. Reg., regulation.

We previously reported on transcriptional data of amnion (n = 7), muscle (n = 6), adrenal (n = 3) and pancreas (n = 7) at W9, W18 and W22 [40] and used this data to test the hypothesis that GOM is associated with the epigenetic downregulation of developmental programs and LOM with upregulation of tissue-specific processes. Genes associated with GOM and involved in embryonic morphogenesis (a process enriched for GOM in all tissues) showed a decrease in transcriptional activity from W9 to W22 in all tissues (amnion, muscle, pancreas: *P* < 0.05; adrenal *P* = 0.87; Fig 4A). In contrast, genes involved in tissue-specific processes found to be enriched for LOM (S3 Table) increased in transcription from W9 to W22 (*P* < 0.05; Figs 4B and S4D).

Altogether, these findings emphasize that DNA methylation dynamics during human fetal development is associated with the availability to transcription of both general embryonic programs (shutting those down for transcription) as well as tissue-specific developmental programs (making those available for transcription).

### Dynamically methylated regions correlate with developmental and tissue-specific genes

**Fig 5:**
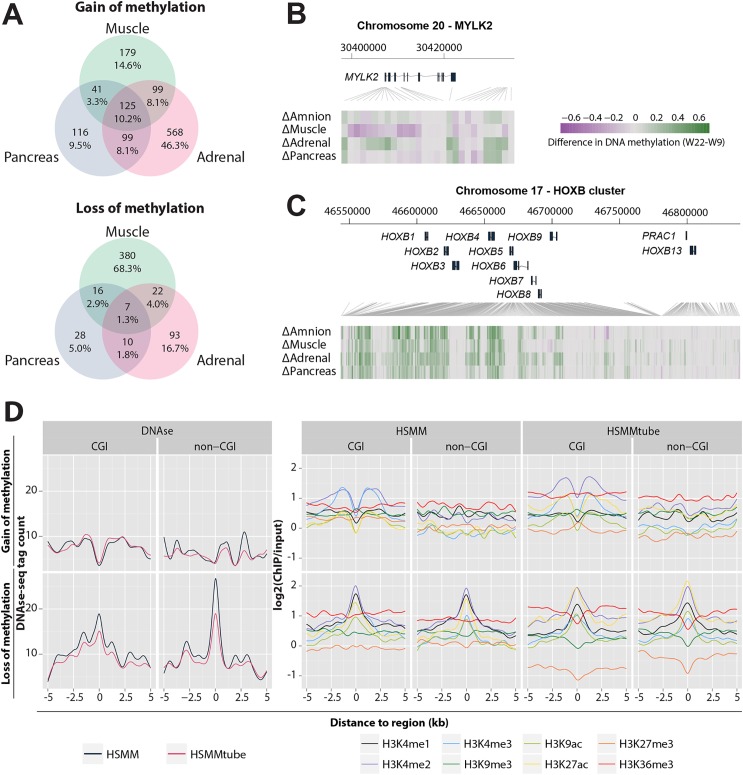
Association of gain and loss of DNA methylation, DNAse I hypersensitive sites and histone modifications. (**A**) Venn diagram visualizing the overlaps between genes with a gain and a loss of methylation of the three embryonic tissues. (**B**) Methylation difference between W9 and W22 of *MYLK2* in the four tissues. (**C**) Methylation difference between W9 and W22 of the *HOXB* cluster. (**D**) Mean DNAse I hypersensitive (DHS) and histone modifications signals in a 5 kb flanking region of the muscle dDMRs in HSMMs and HSMMtubes. HSMM, human skeletal muscle myoblasts; HSMMtube, human skeletal muscle myotubes.

From the dynamically methylated CpGs, we identified 2,229 development-related differentially methylated regions (dDMRs, defined as 3 consecutive differentially methylated CpGs within 1kb of each other) undergoing GOM (amnion 185; muscle 530; adrenal 1,065; pancreas 449) and 1,017 undergoing LOM (amnion 388; muscle 482; adrenal 136; pancreas 61; S4 Table). After mapping the dDMRs to the nearest gene locus we observed that the percentage of common genes in the embryonic tissues showing LOM dDMRs was 1.3%, whereas those showing GOM was 10.2% (Fig 5A).

LOM dDMRs were associated with genes involved in tissue-specific functions such as *MYH3* in muscle (muscle contractile protein), *MC2R* (adrenocorticotropic hormone receptor) in adrenal and *PFKFB3* (involved in insulin secretion) in the pancreas (Table 2 and S5A Fig). As an example of tissue specificity encountered in the dDMRs showing a loss of DNA methylation, we zoomed in on the *MYLK2* locus, a muscle-specific gene [41]. The methylation of the *MYLK2* promoter and first exon in the muscle decreased during development, but increased (or remained constant) in the other organs studied (Fig 5B). Interestingly, GOM dDMRs were associated with (tissue-specific) developmental genes, such as *PAX3* in muscle, a key gene in myogenesis [42], and *NKX6*.*1* in pancreas, an important gene in beta-cell development [43] (Table 2 and S5A Fig), but also near well-known developmental genes including the *HOXB* (Fig 5C) and other *HOX* clusters (S6A Fig) that play a key role in embryonic patterning and morphogenesis [44].

**Table 2:** Table with six genes per tissue highlighting the tissue specificity of the genes found near dDMRs with a loss of methylation as well as the association of dDMRs with a gain of methylation with tissue-specific developmental genes.

	Gain of methylation			Loss of methylation		
Tissue	Gene	Features	Function	Gene	Features	Function
**Amnion**	*TNXB*	GB	Cell adhesion	*TNXB*	PP/GB	Cell adhesion
	*TFAP2A*	IG/DP	Cell differentiation	*DIP2C*	GB	Ectoderm development
	*TFAP2B*	GB	Cell proliferation	*CD59*	PP	Lymphocyte signal transduction
**Muscle**	*SIX3*	IG/DP/GB/DS	Transcription factor in repression of WNT	*MYLK2*	PP	Myosin light chain kinase
	*HLX*	DP/GB/DS	Homeobox TF factor in muscle development	*MYOZ1*	PP	Calcineurin signaling in muscle
	*PAX3*	GB/PP	Muscle development	*MYH3*	PP	Muscle contractile protein
**Adrenal**	*TBX3*	IG/DP/DS	TF in developmental processes	*KCNQ1*	PP	Potassium channel protein
	*NR2F2*	IG/DS	Steroid thyroid hormone nuclear receptor	*MC2R*	PP	Adrenocorticotropin receptor
	*KCNQ1*	PP/GB	Potassium channel protein	*SEC14L1*	PP	Intracellular transport
**Pancreas**	*NKX6*.*1*	DP	Beta cell development	*SLC25A22*	PP	Glucose responsiveness
	*PROX1*	DP	Co-repressor of HNF4A	*FAIM3*	PP	Promotes β-cell proliferation
	*PRDM16*	GB	Transcription factor activity	*PFKFB3*	GB	Insulin secretion

However, when comparing all identified dDMRs to previously identified adult (tissue-specific) tDMRs using the 450k array [6], about 50% of the GOM dDMRs were not identified as tDMRs in adult tissues (S5B Fig), while 32% and 38%, of the LOM dDMRs in muscle and pancreas, respectively, were unique for those fetal tissues (adult data on adrenal was absent). The persistence into adulthood of subsets of GOM and LOM dDMRs was confirmed using WGBS data for adult muscle [25] (S5C Fig). These results suggest that the study of DNA methylation dynamics in fetal development will identify regions that are remodelled during development and are missed when studying adult tissues only.

We further explored the potential biological validity of the 1,012 muscle dDMRs using ENCODE data [24] on human skeletal muscle myoblasts (HSMMs) and their differentiated derivatives, human skeletal muscle myotubes (HSMMtubes). In HSMMs and HSMMtubes, DNAse I hypersensitive sites (DHSs), which mark genomic regions of open chromatin associated with transcriptional activity, were abundant at LOM dDMRs, particularly in CpG-poor regions (CGI-shelves and non-CGI regions, Fig 5D left). DHSs were depleted at GOM dDMRs in CpG-rich regions (CGIs and CGI-shores, Fig 5D left). Consistent with an increased transcriptional activity, LOM dDMRs were also enriched in myotubes and myoblasts (ENCODE [24]) for histone H3 lysine 4 methylation ((H3K4me1, -me2, -me3) [25,45], and acetylation of histone H3 at lysine 9 and 27 (H3K9ac, H3K27ac), all marks associated with active regulatory regions [25,45] (Fig 5D right). In contrast, these active histone modifications were depleted for GOM dDMRs in CpG-rich regions (Fig 5D right). LOM dDMRs were depleted of H3K9me3 (marking inactive DNA), H3K27me3 (marking Polycomb-repressed regions) and H3K36me3 in HSMMtubes but not in their precursor cells HSMMs (Fig 5D right). A final indication for the functional relevance of the muscle dDMRs was that 124 out of 482 LOM dDMRs significantly overlapped (*P <* 0.0001) with binding sites of the muscle-specific transcription factor *MYOD* in HSMMs (188/482 in HSMMtubes), whereas only 7 out of the 530 GOM dDMRs mapped to *MYOD* binding sites[46] (8/530 in HSMMtubes; S5D Fig).

## Discussion

**Fig 6:**
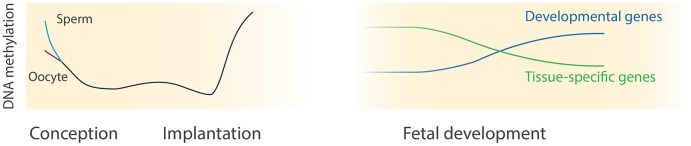
DNA methylation dynamics during human development. This illustration depicts the current comprehensive knowledge of DNA methylation during human pre- and postimplantation development. The knowledge about DNA methylation during human preimplantation (left panel) is derived from [9–12] whereas our study sheds light on postimplantation development (right panel).

Here, we show that human tissues already exhibit a specific DNA methylation signature as early as W9 of fetal development. In addition, the DNA methylation landscape is subjected to considerable changes from the first to second trimester of gestation as the developing organs gain complexity and functionality. Our study highlights that dynamic DNA methylation is not only an integral part of early preimplantation embryo development and implantation [9–11], but continues to be a key feature of epigenetic remodelling during human fetal development. While global changes in levels of DNA methylation characterize development until implantation (Fig 6), these are not observed during fetal development. Instead, distinct LOM occurs near tissue-specific genes and GOM occurs near developmental genes in a largely tissue-independent fashion (Fig 6). Our direct assessment of DNA methylation dynamics suggests that a larger proportion of the methylome is remodelled during development than previously thought [3,6,47].

Interestingly, the functional relevance of identified dynamic regions was further exemplified by the changes in expression of their nearest genes. While the nearest genes of regions gaining DNA methylation associated with embryonic morphogenesis showed loss of expression, the nearest genes of regions losing DNA methylation showed increased expression over time. In agreement with our observations, LOM of hematopoietic-specific genes has been observed during human hematopoietic differentiation *in vitro* [48] and have been linked to transcriptional changes in human T-cell development [49]. Moreover, several mouse and human *in vitro* studies demonstrated that the methylation of developmental genes increases [19,50] and tissue-specific functional genes lose methylation [5,51] during stem and progenitor cell differentiation. Lastly, DNA demethylating agents, such as 5-azacytidine, have been shown to promote stem cell differentiation and maturation of skeletal myotubes in mice [52,53]. Further experimental studies are required to evaluate the mechanistic role of DNA methylation in development.

Although our study reveals general principles of DNA methylation dynamics during human fetal development, it should be noted that a limited number of tissues and individuals was investigated; and that we used a genome-wide method interrogating a relatively small proportion of all CpGs in the human genome. Expansion to more tissues and the application of whole-methylome technologies will lead to a more comprehensive catalogue of regulatory regions. However, by extensive inclusion of external fetal and adult 450k array datasets, we have consolidated our findings. Moreover, the use of external available WGBS data confirmed the results obtained by the 450k array data.

Since we studied organ biopsies similar to previous studies investigating biopsies of human adult tissues [3,15], the methylation profiles we report reflect the average of multiple cell types. The cellular complexity of the organs investigated led to an underestimation of the actual DNA methylation dynamics in individual cell types. This is exemplified by the detection of a considerably larger number of CpGs displaying dynamic methylation in muscle which has an exclusive mesodermal origin in comparison with adrenal and pancreas which are composed by cells originating from two different germ layers (adrenal: mesoderm and ectoderm (neural crest); pancreas: endoderm and mesoderm). However, it is unlikely that the methylation dynamics observed is an epiphenomenon of this cellular complexity instead of being driven by cell differentiation and maturation. This is obvious for genes associated with GOM that appears to be shared across organs to repress general developmental programs during development. In contrast, genes associated with LOM displayed tissue-specific patterns. Their intricate involvement in organ-specific functions was emphasized by tight linkage to biological processes and chromatin states relevant to the organs investigated. Moreover, between W9 and W22, the organs analysed are mainly composed of progenitor cells; perfusion by blood and lymphatic vasculature, and innervation by neural crest cell derivatives still plays a minor role as compared with adult organs (S1A Fig). In the future, single-cell methodology [54,55] will enable comparing single-cell DNA methylomes of the various adult cell types to their fetal progenitor counterparts.

Studies of DNA methylation landscapes of human fetal development may serve as reference in the development of (organoid) differentiation models [56] and, moreover, shed light on potential mechanisms underlying genetic associations and studies in the field of epigenetic epidemiology [57] focussing on the prenatal environment.

## Materials and Methods

### Ethical statement

The Medical Ethical Committee of the Leiden University Medical Center approved this study (P08.087). Informed consent was obtained on the basis of the Declaration of Helsinki (World Medical Association).

### Fetal tissue

Human fetal tissues (amnion, skeletal muscle, adrenal glands, pancreas) at gestational age W9, W18, W22 (S2A Fig) were collected from elective abortion material (vacuum aspiration) without medical indication. In this study, “weeks of gestation” was used as determined by the last menstrual period (LMP). After collection, the material was washed with 0.9% NaCl (Fresenius Kabi, France) and the identified organs were immediately snap-frozen using dry ice and stored at -80°C until further processing. Histology was performed as previously described [58]. The images were taken with an Olympus AX70 microscope (Olympus, Japan) provided with a XC50 digital colour camera (Olympus, Japan).

### DNA extraction

Tissues were homogenized with a pestle and lysed overnight at 56°C with proteinase K (600 mAU/ml, Qiagen, Germany) in ATL buffer (Qiagen, Germany). After lysis, residual RNA in the samples was degraded using RNase A (10 mg/μl, Invitrogen, USA). Subsequently, genomic DNA (gDNA) was extracted on the basis of phenol/chloroform. Briefly, lysates were transferred to Phase Lock Heavy Gel 2ml Eppendorf tubes (5PRIME, Germany) and 700 μl of 25:24:1 Phenol/Chloroform/Isoamyl alcohol was added and spun down for 5 minutes. The aqueous phase was transferred to a Phase Lock tube and the latter step was repeated. The aqueous phase was transferred to a new Phase Lock tube and 700 μl 24:1 Chloroform/Isoamyl alcohol was added and spun down for 5 minutes. The aqueous phase was transferred to a Phase Lock tube and the latter step was repeated. The aqueous phase was transferred to a new 2 μl Eppendorf tube (Eppendorf AG, Germany), 70 μl 3M sodium acetate (Ambion, USA) and 1400 μl ice cold 100% ethanol were added. gDNA was precipitated over night at -20°C. Eppendorf tubes were spun down at 4°C for 15 minutes and washed twice with 70% ethanol. After the pellet was dry, gDNA was solubilized in AE buffer (Qiagen, Germany) and stored at 4°C. DNA concentration was determined using the Qubit dsDNA BR Assay Kit on a Qubit 2.0 Fluorometer (Invitrogen, USA). gDNA was bisulfite converted using the EZ-96 DNA methylation kit (Zymo Research, Orange County, USA) with an average input of 600 ng gDNA. Following bisulfite conversion, DNA methylation data was generated using Illumina HumanMethylation450 BeadChip according to the manufacturer’s protocol.

### (Pre-) processing of the Illumina 450k BeadChip data

All analyses were performed using *R* statistics, version 3.0.1. The 65 polymorphic SNP probes featured on the 450k array were used to exclude potential sample mix ups. Data was imported in *R* using *minfi* [59] and processed and normalized using a custom pipeline: Arrays were removed if they had a low median intensity, a high background signal or with incomplete bisulfite conversion, but none were excluded (S1B, S1C and S1D Fig). The CpGs on chromosome X and Y were used to confirm sex (S1C Fig). Next, probes with a low bead count (< 3), high detection *P*-value (> 0.01), and with a low success rate (< 95%), and ambiguously mapped probes [60] were removed. After probe filtering, all arrays contained > 95% of the original number of probes. Background correction and colour correction were applied and the data was quantile normalized (*lumi* [61]). To adjust for the type I/II bias BMIQ was applied [62]. In our analyses, CpG sites in the sex chromosomes (Y and X) were excluded. High correlation was found between samples from the same time point and tissue but also between time points of the same tissue (S1D Fig). To exclude chromosomal abnormalities, we calculated the copy number aberration based on the signal intensities using the method published by Feber *et al*. [63] as implemented in the *R* package *ChAMP* [64]. From these results, no abnormalities were found in the samples used (S1E Fig).

### Bioinformatics analyses

Multidimensional scaling and clustering was performed based on Euclidean distance. For the DNA methylation over time within features, a genic annotation was combined with a CGI-centric annotation as presented before [6] to determine the median methylation per combined feature. *P* were calculated using quantile regression based on the median (R package *quantreg* [65]). Figures were made using the *R*-packages *ggplot2* [66] and *GenomeGraphs* [67].

*Tissue-specific hypomethylation*: CpGs with a standard deviation ≥ 0.1 within the tissue of interest were discarded. Relative tissue-specific hypomethylation was defined as hypomethylation of the tissue of interest compared to the other tissues, with a difference of ≥ 0.20 in beta value. CpGs sites were selected if a difference was consistent in each of the time points.

*Dynamic methylation*: CpGs with a high standard deviation ≥ 0.10 within time point/tissues indicative of an instable estimate of DNA methylation were discarded from this analysis. Gain and loss of methylation was defined a gain/loss of ≥ 0.20 between W9 and W22 and W18 in between the two time points (W18 was allowed to be 0.05 lower/higher in beta value than W9/W22 respectively). The CpGs with a gain or a loss of methylation were used for the combined genic/CGI-centric annotation and expressed as an odds ratio. Chromatin state segmentation data [25] were downloaded from the Epigenomics Roadmap Project for fetal muscle (W15 female), fetal adrenal (W13 male), amnion (W16 male) and pancreatic islets (adult) and enrichment of dynamically methylated CpGs was calculated.

*DMRs*: In both, the relative hypomethylated CpGs (tHRs) and the CpGs with a gain or a loss of methylation (dDMRs), DMRs were called using an algorithm described before [6]. Briefly, DMRs (tHRs and dDMRs) were defined by three consecutive CpGs that matched a criterion (that is, relative hypomethylation or gain/loss of methylation) with a maximum of 1 kb between CpGs and with at highest three CpGs that did not match the criterion.

*Gene ontology*: Tissue-specific hypomethylated and dynamically methylated CpGs were mapped to their nearest gene (that is to the nearest TSS or TES of a gene) and tested for enrichment of gene ontology terms using DAVID [68]. For the tissue-specific hypomethylation we used a *P* cut-off of 0.05 on the raw *P* as the number of CpGs was relatively low. For the dynamically methylated CpGs a FDR cut-off of 0.05 was set as cut-off for enrichment in GO terms. A background set was used containing nearest genes of all CpGs covered on the array.

*Gene expression data*: Transcriptional data of the four tissues at W9, W18, W22 (amnion: n = 2, 3, 2, muscle: n = 2, 2, 2; adrenal: n = 1, 1, 1; pancreas: n = 3, 2, 2) were used. The counts per million (CPM) expression levels were calculated using the R package edgeR 3.2.4 [69,70]. For the plots, the arithmetic mean of the biological replicates was used and the median of all genes plotted. To access enrichment of up- and downregulation, a probability test was used.

*MYOD*, *DNAse I*, *histone marks and WGBS*: Overlaps between *MYOD* binding peaks and muscle dDMRs were calculated. To test for significance, we calculated an empirical distribution by performing 20,000 permutations with 482 (gain of methylation) and 530 (loss of methylation) DMR-like regions each and determined the overlap with the *MYOD* binding sites. DMR-like regions were defined as regions with equal characteristics as dDMRs identified: an inter-CpG distance smaller than 1 kb and an average length of five CpGs per DMR-like region (n~8 x 10^4^ regions). The two-sided *P* was determined using the empirical distribution.

DNAse I and histone mark data of human skeletal muscle myoblasts (HSMMs) and human skeletal muscle myotubes (HSMMtubes) were downloaded from the ENCODE website [24]. DNAse I hypersensitivity was expressed as the count of DNAse-seq tags. The enrichment of histone marks was expressed as the log2 of the ChIP/input. The total number of reads within the myotubes was different from the total number of reads in the myoblast data and, therefore, the data was normalized. dDMRs were classified as island (CGIs and their shores) or non-island dDMRs, and DNAse-seq tags and histone marks around dDMRs were mapped up to 5 kb up- and downstream.

CpG sites of WGBS data were mapped to hypomethylated and dynamic regions and their 5kb flanking regions. Using a smooth spline, the methylation around the regions was smoothed for the adult and fetal data.

### Accession numbers

Methylation data has been deposited in the NCBI’s Gene Expression Omnibus [71] under accession number GSE56515. External datasets that have been used in this manuscript include: fetal and adult DNA methylation data of various tissues from Nazor *et al*. (Gene Expression Omnibus (GEO) accession number: GSE31848) [15], fetal brain DNA methylation data from Spiers *et al*. (GEO accession number: GSE58885) [22], fetal liver DNA methylation data from Bonder *et al*. (GEO accession number: GSE61279) [30], adult DNA methylation data of various tissues from Slieker *et al*. (GEO accession number: GSE48472) [6], fetal Deep SAGE expression data of the four tissues studied here from Roost *et al*. (GEO accession number: GSE66302) [40], adult DNA methylation brain data from Pidsley *et al*.(GEO accession number: GSE61431) [38], WGBS data of fetal and adult muscle generated by the Epigenomics Roadmap consortium (GEO accession numbers: GSM1172596 and GSM1010986), MYOD binding peaks from MacQuarrie *et al*. (GEO accession numbers: GSM1218849 and GSM1218850) [46].

## Supporting Information

S1 Fig Quality control. (**A**) Histology of the four tissues used in this study during development stained with Haematoxylin and Eosin (H&E). White arrow points to first trimester muscle. Scale bars: 100 μm. (**B**) Density plot of the data per sample coloured by tissue. (**C**) Density plot of the sex chromosomes. (**D**) Pearson correlation between the biological replicates. The highest correlation was found between tissues and time points. (**E**) Assessment of chromosomal abnormalities.
(JPG)

S2 Fig General description of tissue-specific DNA methylation signatures. (**A**) Table of the fetal samples included in this study. (**B**) Multidimensional scaling based on Euclidean distance, from left to right: coloured by tissue, time point and individual. (**C**) Autosomal DNA methylation in three classes (0%-25%; 25%-75%, 75–100%). (**D**) DNA methylation of the X chromosome in female samples. **(E)** Hierarchical clustering of the current data with external fetal data of various tissues [15,22,30].
(JPG)

S3 Fig Tissue-specific hypomethylated CpGs and tHRs. (**A**) The odds ratios of hypomethylated CpGs per tissue in a combined genic and CGI-centric annotation (Fig 2B). (**B**) The odds ratios of hypomethylated CpGs per tissue in the chromatin state segmentations of amnion, fetal muscle, fetal adrenal and adult pancreatic islets (Fig 2C). (**C**) Comparison of hypomethylated CpGs per tissue in fetal and adult external data [6,15]. (**D**) Heatmap representing DNA methylation levels of identified tHRs in amnion, muscle and pancreas in Table 1. (**E**) WGBS DNA methylation profile near hypomethylated regions in muscle of fetal against adult muscle [25].
(JPG)

S4 Fig Dynamic DNA methylation during development. (**A**) Mean methylation of CpGs with a gain or loss of DNA methylation for fetal tissues and their adult counterpart, including fetal brain and fetal liver [6,15,22,38]. (**B**) The enrichment of dynamically methylated CpGs in a combined genic and CGI-centric annotation (Fig 3C), significant odds ratios (Chi-squared test *P* < 0.05) are depicted in black. (**C**) The enrichment of dynamically methylated CpGs in the chromatin state segmentations of amnion, fetal muscle, fetal adrenal and adult pancreatic islets (Fig 3D). (**D**) Expression profiles of genes near dynamic regions with loss of methylation grouped by the Gene Ontology terms for each of the four tissues from S3 Table [40].
(JPG)

S5 Fig Dynamic CpGs cluster into development-related DMRs. (**A**) Average DNA methylation levels of the genes from Table 2. (**B**) Overlap between identified dynamic and hypomethylated regions per tissue and adult tDMRs expressed as percentage overlap. (**C**) WGBS DNA methylation profile near regions with gain and loss of methylation in muscle of fetal against adult muscle [25]. (**D**) Number of *MYOD* binding sites relative to the dynamic regions identified in HSMMs and HSMMtubes. HSMMtube, human skeletal muscle myotube.
(JPG)

S6 Fig Dynamic DNA methylation in the HOX clusters. (**A**) DNA methylation patterns in the four developmental HOX clusters HOXA, HOXB, HOXC and HOXD. The bottom heatmap of each cluster zooms in on a smaller genomic region.
(JPG)

S1 Table GO enrichment of hypomethylated CpGs per tissue. (XLSX)

S2 Table Genes associated with tHRs. (XLSX)

S3 Table GO enrichment of CpGs with a gain/loss of DNA methylation. (XLSX)

S4 Table Genes associated with dDMRs with gain and loss of DNA methylation. (XLSX)
