# Supplementary figures and images for: DNA Methylation Landscapes of Human Fetal Development
Source: PLoS Genet. 2015 Oct 22;11(10):e1005583. doi: 10.1371/journal.pgen.1005583 (PMC4619663; doi:10.1371/journal.pgen.1005583)

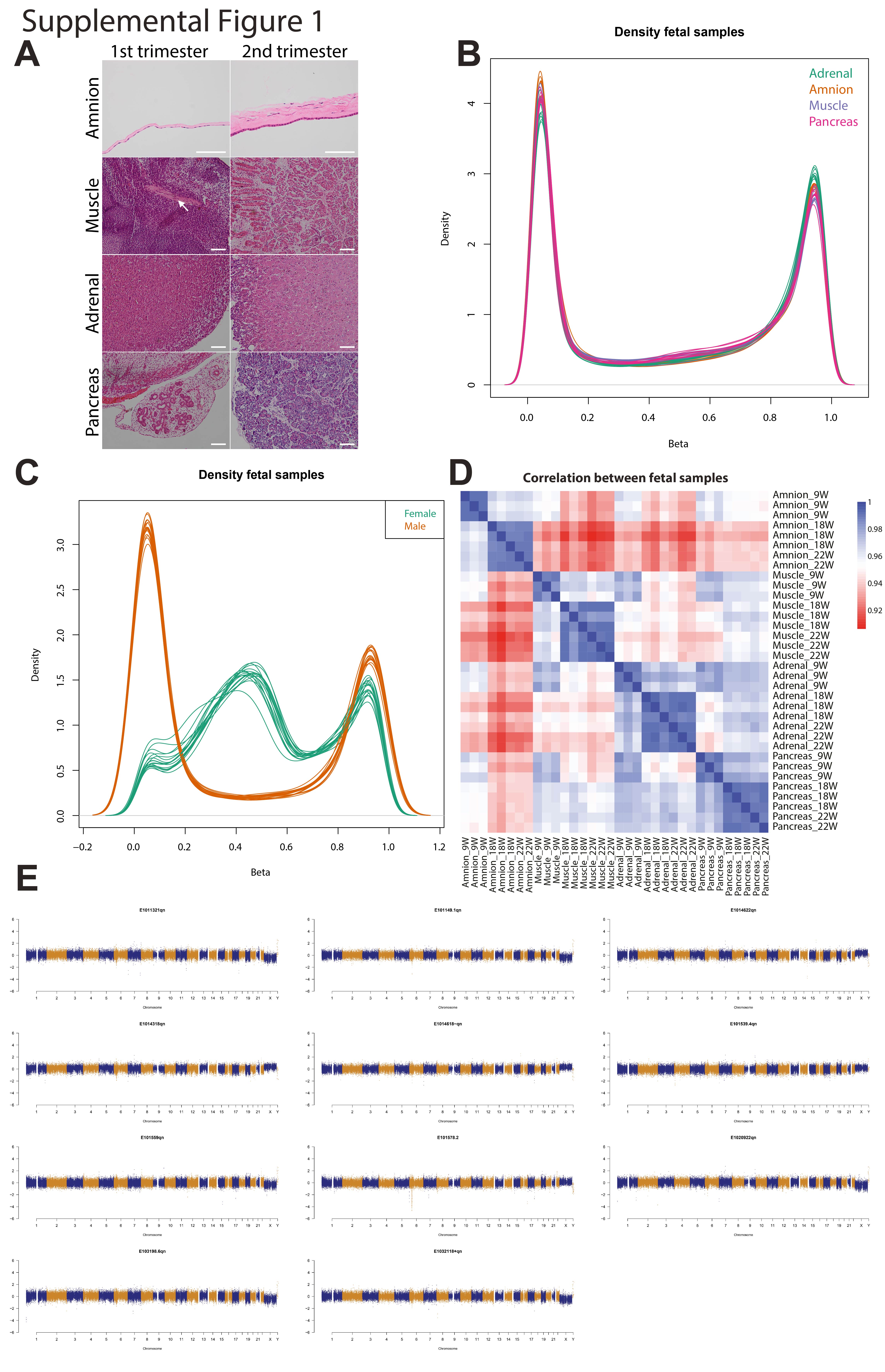

Supplement: S1 Fig — (A) Histology of the four tissues used in this study during development stained with Haematoxylin and Eosin (H&E). White arrow points to first trimester muscle. Scale bars: 100 μm. (B) Density plot of the data per sample coloured by tissue. (C) Density plot of the sex chromosomes. (D) Pearson correlation between the biological replicates. The highest correlation was found between tissues and time points. (E) Assessment of chromosomal abnormalities. (JPG) [file pgen.1005583.s001.jpg]

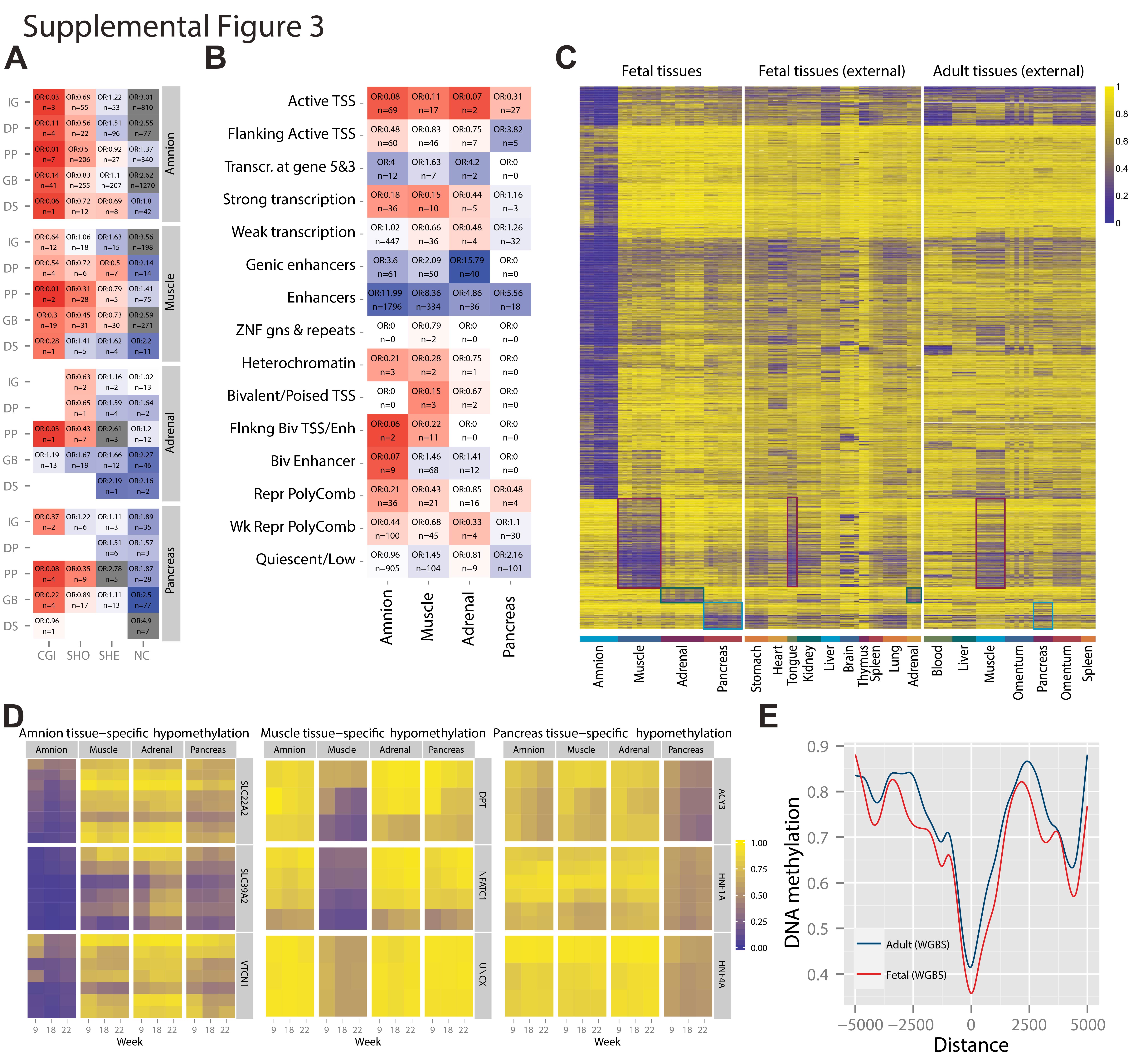

Supplement: S3 Fig — (A) The odds ratios of hypomethylated CpGs per tissue in a combined genic and CGI-centric annotation (Fig 2B). (B) The odds ratios of hypomethylated CpGs per tissue in the chromatin state segmentations of amnion, fetal muscle, fetal adrenal and adult pancreatic islets (Fig 2C). (C) Comparison of hypomethylated CpGs per tissue in fetal and adult external data [6,15]. (D) Heatmap representing DNA methylation levels of identified tHRs in amnion, muscle and pancreas in Table 1. (E) WGBS DNA methylation profile near hypomethylated regions in muscle of fetal against adult muscle [25]. (JPG) [file pgen.1005583.s003.jpg]

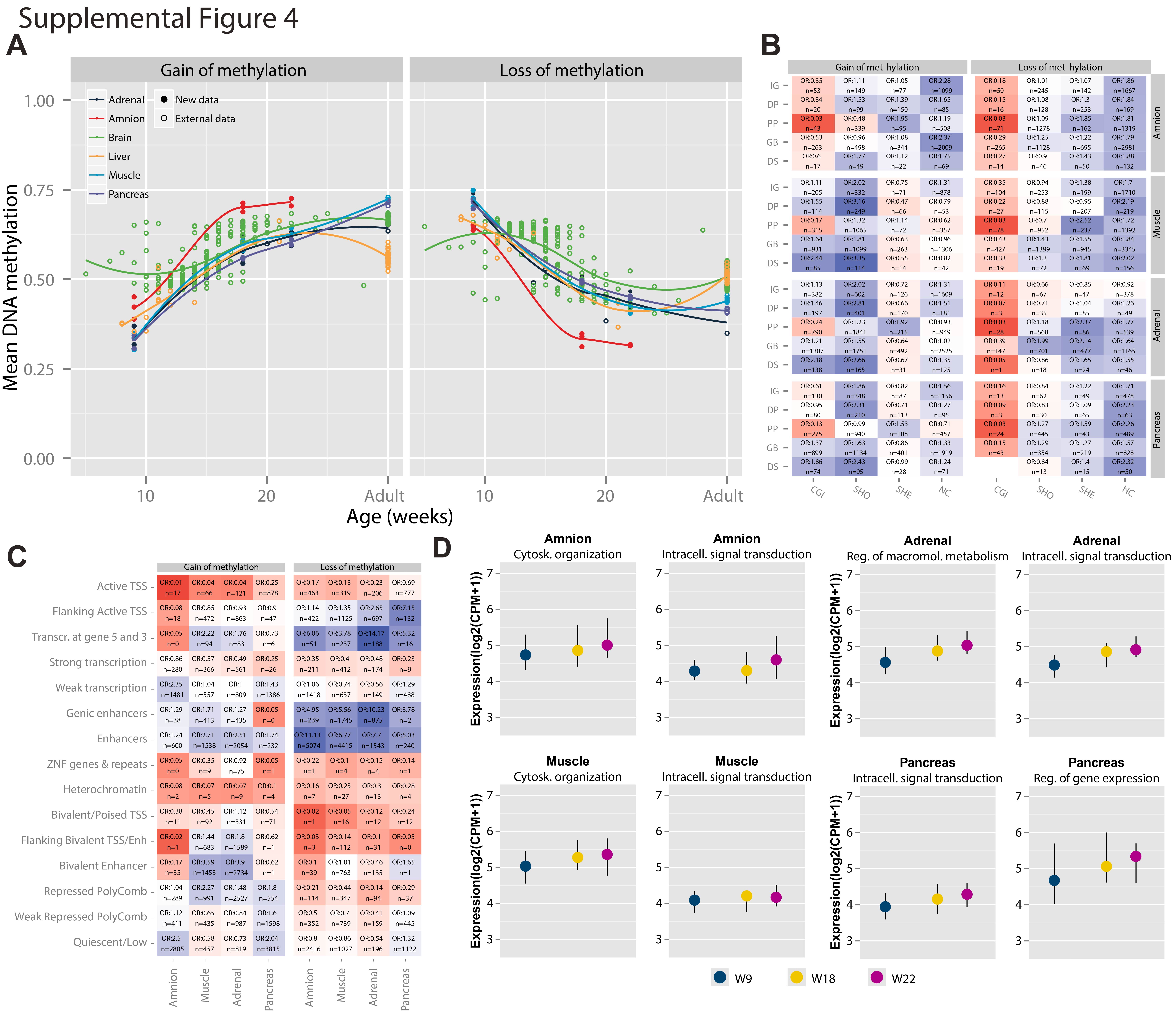

Supplement: S4 Fig — (A) Mean methylation of CpGs with a gain or loss of DNA methylation for fetal tissues and their adult counterpart, including fetal brain and fetal liver [6,15,22,38]. (B) The enrichment of dynamically methylated CpGs in a combined genic and CGI-centric annotation (Fig 3C), significant odds ratios (Chi-squared test P < 0.05) are depicted in black. (C) The enrichment of dynamically methylated CpGs in the chromatin state segmentations of amnion, fetal muscle, fetal adrenal and adult pancreatic islets (Fig 3D). (D) Expression profiles of genes near dynamic regions with loss of methylation grouped by the Gene Ontology terms for each of the four tissues from S3 Table [40]. (JPG) [file pgen.1005583.s004.jpg]

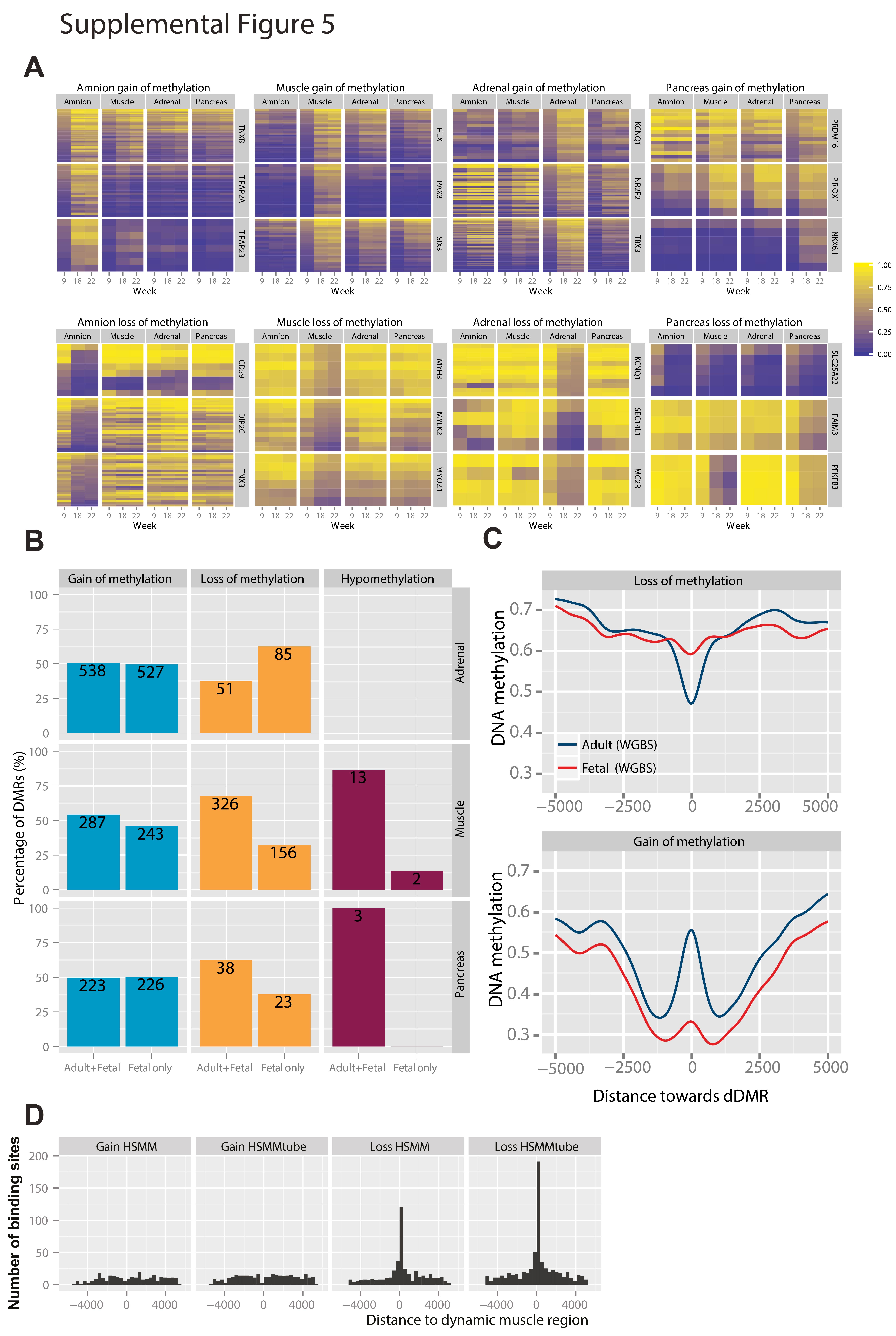

Supplement: S5 Fig — (A) Average DNA methylation levels of the genes from Table 2. (B) Overlap between identified dynamic and hypomethylated regions per tissue and adult tDMRs expressed as percentage overlap. (C) WGBS DNA methylation profile near regions with gain and loss of methylation in muscle of fetal against adult muscle [25]. (D) Number of MYOD binding sites relative to the dynamic regions identified in HSMMs and HSMMtubes. HSMMtube, human skeletal muscle myotube. (JPG) [file pgen.1005583.s005.jpg]

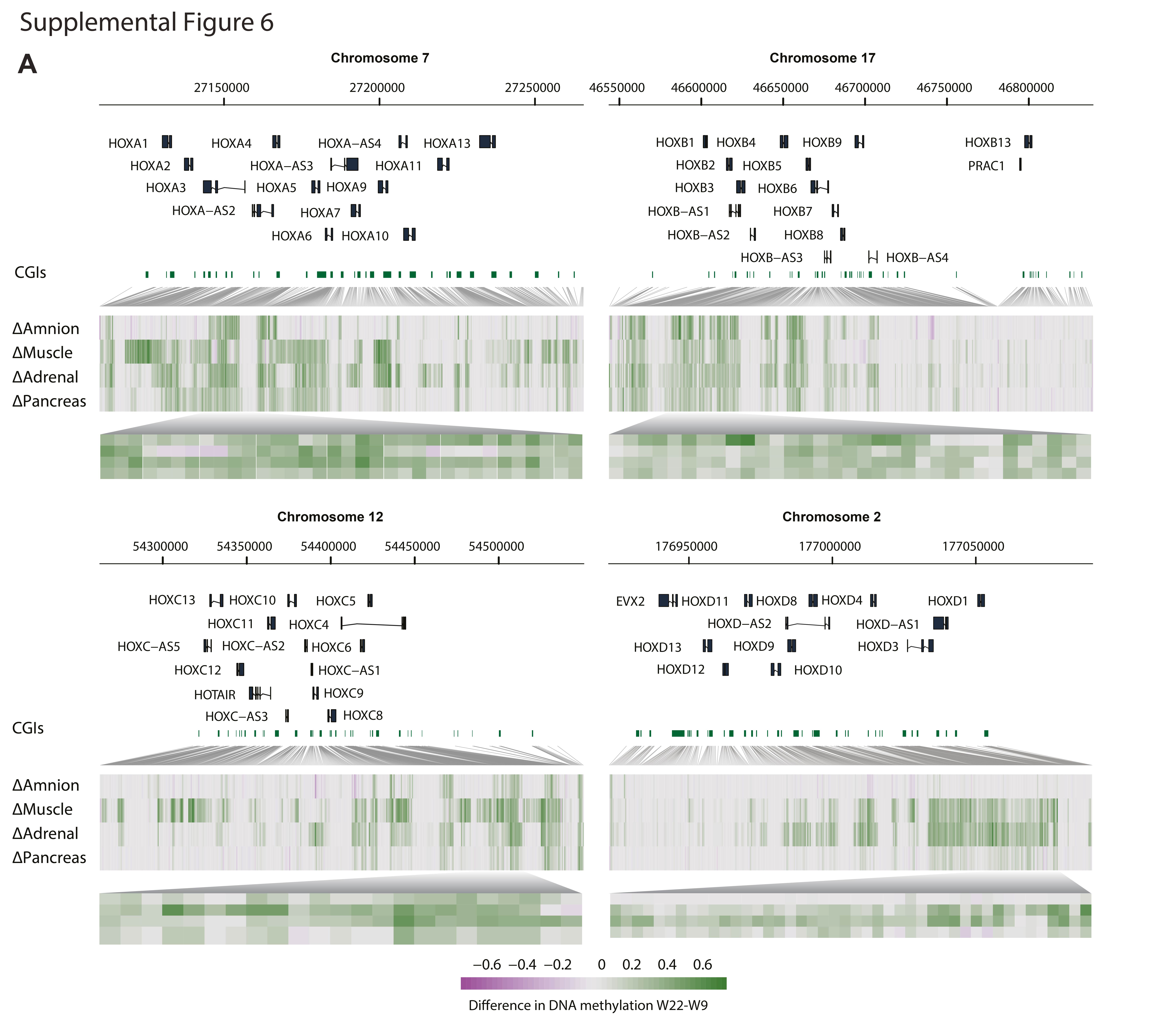

Supplement: S6 Fig — (A) DNA methylation patterns in the four developmental HOX clusters HOXA, HOXB, HOXC and HOXD. The bottom heatmap of each cluster zooms in on a smaller genomic region. (JPG) [file pgen.1005583.s006.jpg]
